# Factors affecting missed nursing care in hospitalized frail older adults in the medical wards: a qualitative study

**DOI:** 10.1186/s12877-021-02524-z

**Published:** 2021-10-14

**Authors:** Zahra Rezaei-Shahsavarloo, Foroozan Atashzadeh-Shoorideh, Abbas Ebadi, Robbert J. J. Gobbens

**Affiliations:** 1 Student Research Committee, School of Nursing and Midwifery, Shahid Beheshti University of Medical Sciences, Tehran, Iran; 2 Department of Psychiatric Nursing & Management, School of Nursing and Midwifery, Shahid Labbafinezhad Hospital, Shahid Beheshti University of Medical Sciences, Tehran, Iran; 3 Behavioral Sciences Research Center, Life Style Institute, Baqiyatallah University of Medical Sciences, Tehran, IR Iran; 4 Nursing Faculty, Baqiyatallah University of Medical Sciences, Tehran, IR Iran; 5 Faculty of Health, Sports and Social Work, Inholland University of Applied Sciences, Amsterdam, the Netherlands; 6 Zonnehuisgroep Amstelland, Amstelveen, The Netherlands; 7 Department of Family Medicine and Population Health, Faculty of Medicine and Health Sciences, University of Antwerp, Antwerp, Belgium

**Keywords:** Frailty, Frail Older Adults, Hospitalization, Missed Nursing Care, Qualitative Study

## Abstract

**Background:**

Frail older adults who are hospitalized, are more likely to experience missed nursing care (MNC) due to high care needs, communication problems, and complexity of nursing care. We conducted a qualitative study to examine the factors affecting MNC among hospitalized frail older adults in the medical units.

**Methods:**

This qualitative study was carried using the conventional content analysis approach in three teaching hospitals. Semi-structured interviews were conducted with 17 nurses through purposive and snowball sampling. The inclusion criteria for the nurses were: at least two years of clinical work experience on a medical ward, caring for frail older people in hospital and willingness to participate. Data were analyzed in accordance with the process described by Graneheim and Lundman. In addition, trustworthiness of the study was assessed using the criteria proposed by Lincoln and Guba.

**Results:**

In general, 20 interviews were conducted with nurses. A total of 1320 primary codes were extracted, which were classified into two main categories: MNC aggravating and moderating factors. Factors such as “age-unfriendly structure,” “inefficient care,” and “frailty of older adults” could increase the risk of MNC. In addition, factors such as “support capabilities” and “ethical and legal requirements” will moderate MNC.

**Conclusions:**

Hospitalized frail older adults are more at risk of MNC due to high care needs, communication problems, and nursing care complexity. Nursing managers can take practical steps to improve the quality of care by addressing the aggravating and moderating factors of MNC. In addition, nurses with a humanistic perspective who understand the multidimensional problems of frail older adults and pay attention to their weakness in expressing needs, can create a better experience for them in the hospital and improve patient safety.

## Background

All over the world, the population of older adults is growing as a result of health policy implementation. This increase has caused serious concerns for the health care system [1]. One of the most difficult problems of aging is the clinical condition of frailty. This problem is characterized by a decrease in physiological reserves in the face of stressors [2]. Frailty is theoretically defined as “a state of increased vulnerability with decreased physical reserves and loss of function in multiple body systems” [3]. It results from an accumulation of abnormal characteristics that include physical impairment, cognitive impairment, depressive symptoms, decreased functionality, multi-morbidity, malnutrition, and social isolation [4]. In addition, frailty was defined as meeting three of five phenotypic criteria consisting of low grip strength, low energy, slow waking speed, low physical activity, and unintentional weight loss [5]. Frailty affects more than 60% of older adults living in the community [6]. In addition, the prevalence of frailty among hospitalized frail older adults is approximately 48 to 80% [2]. Frailty is associated with adverse events such as falls, fractures, decreased physical function, cognitive impairment, and hospitalization for older adults [7]. Hospitalization imposes a high level of stress on older adults due to inappropriate ward environment [8], disorganized care, increased medication consumption [9], age discrimination [10] and missed nursing care [11]. This process aggravates their frailty, worsens the disease and leads to death [9].

Missed nursing care (MNC) is one of the most important factors contributing to inappropriate care of hospitalized older adults [12]. MNC refers to any aspect of required patient care that is omitted or delayed (either in whole or in part) [13]. Evidence suggests that more than 70% of nurses have MNC, with several factors playing a role [14, 15]. Studies in Mexico [16], Iran [14], and Italy [17] have identified high patient load, nursing staff shortage, high patient turnover, and lack of equipment as the factors that have a greatest impact on MNC [14, 16]. In addition, a scoping review study conducted by Gustafsson et al. (2020) found an association between MNC and patient-reported adverse events and patient-perceived adequacy of staffing, with staff shortages and inadequate experience being the main factors in patient perceptions [18].

The NMC phenomen is more common in frail older adults who are hospitalized [19]. This is because they have high care needs due to their frailty and are unable to perform some activities of daily living [20]. Moreover, communication disorders, depression or social isolation prevent them from expressing their needs [21]. Therefore, special attention must be paid to this phenomenon in hospitalized frail older adults [22]. Despite numerous studies that have been conducted on MNC, no specific study has been found that identifies the factors that influence MNC in this target population. Previous studies have used quantitative measures to identify the factors that impact MNC and have failed to consider the characteristics and care needs of hospitalized frail older adults. Another limitation of previous research is its exclusive focus on aggravating factors and neglect of moderating elements.

Due to the limitations of previous studies identifying the factors contributing to MNC in hospitalized frail older adults and the importance of MNC to patient safety, the present study aimed to explore the factors affecting missed nursing care in hospitalized frail older adults on the medical wards through a qualitative approach.

## Methods

### Study design

This qualitative study was conducted using the conventional content analysis approach. This approach is a normatively appropriate method for discovering in-depth information about participants and describing the quality of a phenomenon [23]. Throughout the research process, the recommendations for qualitative research according to Consolidated criteria for reporting qualitative research (COREQ) were followed [24] (Additional file 1).

Researchers immerse themselves in the data to allow new insights to emerge, also described as inductive category development. This helps in bringing out the subjective truths about the meaning and utterances of the participants [25]. It also helps to better understand the challenges faced by nurses in medical wards in caring for frail elderly adults in Iran.

### Study setting

The study was conducted between August 2019 and July 2020 in the three 500-bed teaching hospitals affiliated with Shahid Beheshti University of Medical Sciences in Tehran, Iran. These hospitals are a tertiary hospital and a referral center. The study focused on the medical departments. These have a capacity of 40 to 45 beds.

### Participants

Two forms of recruitment were used in the present study. First, a purposive sampling of nurses reasonably familiar with frailty and MNC was conducted. Second, a snowballing approach was used to supplement the purposive sampling and expand the recruitment pool [26, 27]. Sampling continued until the researchers reached data saturation to complete the categories and subcategories and to answer the study questions.

The researchers aimed to achieve maximum diversity within the sample [28]. Therefore, the sample was selected using the principle of maximum variation to obtain a diverse study population that would vary in terms of gender, agegroups, shift work, years of work experience, contract status, and education level (Table 1).

**Table 1:** Interviews participant characteristics (*N* = 17)

Participant number	Age (year)	Gender	Shift work	Contract status	Educational level	Work experience (year)
1	37	Female	Morning	Permanent	MSN	14
2	44	Female	Morning	Permanent	MSN	20
3	25	Female	Afternoon	Educational commitment	BSN	5
4	38	Female	Night	Permanent	BSN	15
5	27	Female	Afternoon	Temporary	MSN	4
6	57	Male	Morning	Permanent	MSN	35
7	55	Female	Morning	Permanent	BSN	28
8	36	Female	Afternoon	Permanent	BSN	13
9	33	Female	Afternoon	Permanent	MSN	8
10	33	Female	Afternoon	Permanent	BSN	9
11	41	Female	Night	Temporary	MSN	9
12	39	Male	Morning	Permanent	PhD	13
13	44	Male	Morning	Permanent	PhD	18
14	29	Male	Night	Educational commitment	MSN	2
15	32	Female	Afternoon	Temporary	MSN	5
16	35	Female	Morning	Permanent	BSN	27
17	45	Female	Night	Permanent	MSN	20

*BSN* Bachelor of Science in Nursing; *MSN* Master of Science in Nursing; *PhD* Doctor of Philosophy.

The inclusion criteria for the nurses were: at least two years of clinical work experience in the medical ward, caring for frail elderly in the hospital and willingness to participate. Based on the interviewer’s experience and sound judgment, potential participants were approached at different hospitals and work shifts.

Seventeen interviews were conducted face-to-face. Three follow-up interviews were conducted by telephone, for a total of 20 interviews. The follow-up interviews were conducted to clear doubts and obtain supplementary data. In addition, one of the nurses declined to participate in the study due to her heavy workload.

### Data Collection

Data collection was through semi-structured interviews conducted by a researcher (ZRS) The interviewer had previous experience in conducting individual interviews as part of her academic and professional training. She also has experience of caring for frail older people in hospital as a nurse and family cargiver. She is also interested in for the care of older adults. The interviewer had no previous contact with the participants. In addition, participants identified the interviewer as a doctoral student in nursing who was conducting research for her dissertation.

Interviews lasted between 25 and 80 min, depending on the willingness and tolerance of the participants. The time and place of the interviews were chosen according to the participants’ priorities in a private and comfortable space. All interviews were conducted individually without the presence of other participants in a private room or training room.

The interviews were conducted based on the interview guide (Table 2). The interviews mainly focused on perceptions, attitudes, and their experiences of MNC in taking care of hospitalized frail older adults and the factors that influence them. The interviews began with a series of open-ended questions followed by scoping questions to clarify responses and elicit supplementary data. At the beginning, the interviewer introduced herself to the participants. They were then informed about the research objectives, the method, the non-commercial origin of the sponsor, the audio recording of the interviews, and the possibility of an additional interview if further information was needed. In addition, confidentiality of all information and voices disclosed was assured and the voluntary nature of participation was emphasized. Written informed consent was subsequently obtained from all participants. Also, the interviewer recorded all interviews. She noted the nonverbal communication immediately after the interview.

**Table 2:** interview guide for participants

Introduction	- Short description of the research project (the purpose, form of the interview and definition of frailty) - Consent confirmed - Tell me a little about yourself
Opening questions	- What does a typical shift for you? - Explain your experiences in caring for frail older adults? - Explain your experiences in missed nursing care for frail older adults? - What factors aggravate missed nursing care? - What factors moderate missed nursing care? - What people, resources, and facilities could help you in this situation?
Closing and thanks for participation	- Do you have any additional information or questions you would like to add?

To ensure that the participants understood the concept of frailty in the same way, it was defined using standard definition before the interview began. We used the phenotype of frailty approach to define [29]. To assess the nurses’ perceptions of frailty, they were presented with a case, and asked to identify frailty.

### Data Analysis

The data were analyzed using the conventional method of content analysis according to the process described by Graneheim and Lundman (2004) [30]. Accordingly, we took the following five steps to analyze the data: 1. transcribing the entire interview immediately after it was conducted; 2. reading the entire transcript to obtain a general understanding of it; 3. identifying units of meaning and primary codes; 4. categorizing similar codes into major categories; and 5. identifying the major themes of the categories [30]. MAXQDA 2007 software was used to organize and manage the data.

The interviews were recorded, carefully listened to, transcribed word by word at the first time to establish a link between the data and the participants’ feelings. Then the interviewer reviewed the transcript and made field notes about her initial impressions. During this process, code labels emerged that reflected more than one key thought. These often came directly from the text and produced the initial coding scheme. Codes that were conceptually similar were clustered together and semantically related clusters were then organized into themes. Two experts in qualitative analysis and subject matter (FAS and AE) conducted the peer review and confirmed the codes and themes. In the event of disagreement and differing interpretations among the researchers, several panels consisting of the entire research team reviewed the coding process to agree on a final version.

### Rigor and trustworthiness

The trustworthiness of the study was assessed using the criteria proposed by Lincoln and Guba [31]. To increase credibility, we used member-check and a longer engagement by the researcher. Member-checking of transcripts and findings was conducted with four participants to ensure accuracy. In addition, a longer engagement of the researcher (13 months) was achieved to confirm in-depth understanding of the subject, prevent the collection of improper information, and confirm data saturation while formulating the main categories. As well, to improve the credibility of the results, maximum variation in sampling regarding participants’ age, gender, level of education, and work experience was considered. To enhance confirmability and dependability, an external audit was used. During the external audit, the correctness of the analysis was validated by two experienced qualitative researchers. In addition, to ensure transferability, the participants and the context of the study were described in detail.

## Results

In general, 20 interviews were conducted with nurses. A total of 1320 primary codes were extracted, which were classified into two main categories: MNC aggravating and moderating factors (Table 3). Then, the categories and subcategories were described with quotes from the participants.

**Table 3:** Categories and sub-categories related to Missed Nursing Care of frail older adults

Main categories	Categories	Sub-categories	Quotations
MNC aggravating factors	Age-unfriendly structure	Inappropriate care environment	“This ward is not specifically for older adults. We have a lot of critically ill patients who need urgent care. Sometimes we have patients who need cardiopulmonary resuscitation (CPR). When I have a patient that sick, I do not have time to communicate with older adults and address their needs” (Nurse 5)
		Management defect	“In the clinical assessment forms, there is no specific for older patients. So we cannot distinguish between frail older adults to pay special attention to them” (Nurse 4)
		Discriminatory behaviors	“Usually the critically ill older adults are admitted to the terminal rooms of the ward. We put young patients in our priority, and they are admitted to the rooms near the nursing station.” (Nurse 14)
		Staff’s high workload	“Frail older adults are usually ordered a syrup from MOM because of persistent constipation. If I give the patient the syrup several times a day, we do not have enough aides to help the patient go to the bathroom or change a diaper. So we do not have to give the medicine,” (Nurse 9)
	Inefficient care	Weakness of interdisciplinary care	“Many frail older adults need physiotherapy, occupational therapists, or social help to get back into the community, but many of them are homeless. We actually know our patients need it, but we really can not do anything about it,” (Nurse 5)
		Clinical incompetence	“Novice nurses do not know that they should follow up on the frail older adult’s medications that he is taking at home. For example, an older adult is admitted to the hospital for a fractured pelvic and does not remember his medications. The nurse forgets to track what he’s taking, so the patient does not take the blood pressure pill, blood pressure rises to 20 or 24, and he suffers a brain hemorrhage.” (Nurse 9)
		Weakness in continuing education	“Most of the teaching materials are related to diseases, such as the management of hypertension, diabetes, and special diseases. As far as I know, we do not have special training for aging,” (Nurse 7)
	Frailty of the older adults	Physical health issues	“They cannot manage most daily activities of life on their own and are often dependent,” (Nurse 2)
		psychological health issues	“My patient had severe bleeding in her stool, but she did not tell us. She generally communicates with difficulty, she is mostly depressed.” (Nurse 15)
		Weakness of social support	“Some frail older adults do not have a family caregiver to help them, and sometimes we do not come to help them. Sometimes their needs are ignored until someone like family caregiver can help them. Some of them do not have a home when they are discharged from the hospital and they stay here so the social worker can find a place,” (Nurse 2)
MNC moderating factors	Support capabilities	Supervisor’s support and supervision	“Some of our supervisors do everything they can to solve problems and keep working.” (Nurse 3)
		Active family participation	“A good and active family caregiver is very good for the frail older adult. For example, he moves him, gives him food, gives him medication, helps him to the toilet or bathroom, helps him eat, gives him high-fiber food, gives the patient a sense of familiarity and trust.” (Nurse 9)
	Ethical and legal requirements	Humanistic view to patient care	“Caring for the elderly reminds me of my mother, who was once in the hospital and preferred to be cleaned every day. So I force the nurse aid to bathe the frail elderly or wash their hands and face every shift” (Nurse 3)
		Concerns for being accountable	“If you do not do your job properly, you will be known on the ward as someone who does not take good care of the patients. So I am careful about caring for patients, especially frail older adults and patients without family caregivers.” (Nurse 6)

### MNC aggravating factors

The categories of “Age-unfriendly structure”, “Inefficient care,” and “Frailty of older adults” were presented as aggravating factors for MNC.

#### Age-unfriendly structure

The medical ward does not have a suitable structure for frail older adults. This category consists of the following subcategories: “Inappropriate care environment,” “Management defects,” “Discriminatory behaviors,” and “High staff workload.”

##### Inappropriate care environment

The layout of wards and their psychological environment are considered as the factors contributing to MNC. The long distances between the patient rooms, nurse stations and medication rooms mean that nurses spend a lot of time walking around the ward and less time at the patient’s bedside. Besides, the medical ward environment is crowded and disrupts the nurses in delivering care. “There is a long corridor in the ward which makes it difficult for me to check on my elderly patient because I have to walk fora long time. There are so many students, physicians, and family caregivers here causing chaos, messing up the patient records and confusing me to what I wanted to do,” said, a nurse with a master’s degree and 14 years of clinical work experience (Nurse 1).

Nor is nursing care is not provided exclusively to older adults in medical wards. Patients of different ages and with different diagnoses are admitted, sometimes requiring vital nursing interventions. This causes nursing care to be diverted from older adults and the MNC. A nurse with four years’ experience said (Nurse 5), “This ward is not specifically for older adults. We have a lot of critically ill patients who need urgent care. Sometimes we have patients who need cardiopulmonary resuscitation (CPR). When I have a patient that sick, I do not have time to communicate with older adults and address their needs.”

##### Management defects

Managers of organizations may ignore frail older adults and their needs for the following reasons: 1) incompetence in establishing care guidelines, 2) evaluation based on nonclinical criteria, 3) lack of attention to nurses’ job motivations, and 4) paternalistic approach to planning. They failed to consider the frail state of older adults in care guidelines and assessment protocols. “ In the clinical assessment forms, there is no specific for older patients. So we cannot distinguish between frail older adults to pay special attention to them”(Nurse 4),. Moreover, indirect nursing care is given priority in evaluating nurses’ performance. Therefore, nurses pay more attention to indirect care than direct care at the patient’s bedside. A nurse with four years of experience said (Nurse 5), “The most important thing here is a comprehensive and detailed nursing report. If the first thing that is assessed is the nursing report, I try to spend most of my time and attention on writing the report.

The managers of the organization do not pay attention to the nurses’ job motivations and their opinion in preparing the nursing care plan. They do not consider motivators to improve the quality of care. “If I have to do everything they order and they do not differentiate between someone who has done an excellent job and someone who is sloppy in their care, I am unmotivated to try to do better and provide complete care,” stated a nurse with five years of experience (Nurse 15).

##### Discriminatory behaviors

Medical personnel have negative attitudes toward frail older adults. This attitude is due to the high level of care required, communication problems, and the complexity of care. As a result people are less willing to spend time caring for frail older adults. A male nurse with two years’ experience explained (Nurse 14), “Usually the critically ill older adults are admitted to the terminal rooms of the ward. We put young patients in our priority, and they are admitted to the rooms near the nursing station.”

In addition, many nurses have no hope for treating frail older adults and feel that caring for these patients is pointless. Therefore, they prefer to spend their time with other patients who have more hope for treatment and life. A male nurse with 13 years of experience said (Nurse 12), “When the resuscitation team realizes that the patient is a frail older adult, they say he does not want to continue resuscitation and quickly end it quickly. Our nurses say what good is resuscitation if the patient is not alive?”

##### Staff’s high workload

Nurses feel that numerous non-nursing related tasks such as infection control responsibilities and various indirect tasks such as writing the nursing report lead to MNC. A nurse with four years of experience commented (Nurse 5), “I have to write 28 nursing reports for my 14 patients in an evening night shift. Admitting of new patients, writing medication cards, and all the administrative tasks like filling out charts keep me away from my patients bedside.”

Moreover, the shortage of nurses exacerbates the burden on caregivers and worsens the MNC. “Frail older adults are usually ordered MOM suspension because of persistent constipation. If I give the patient the syrup several times a day, we do not have enough aides to help the patient go to the bathroom or change a diaper. So we do not have to give the medicine,” said a nurse with eight years of experience (Nurse 9).

#### Inefficient care

Nurses are unable to care for frail older adults because for reasons such as “weakness in interdisciplinary care,” “clinical incompetence,” and “weakness in continuing education.”

##### Weakness in interdisciplinary care

Members of the health care team, including physicians, nurses, physical therapists, and dietitians, have a negative attitude toward teamwork and are not experts on aging issues. As a result, they focus on treating the chief complaints of frail older adults and neglect the needs associated with frailty. A female geriatric nurse said (Nurse 17), “An elderly patient was hospitalized for a fall, but the doctor was only looking for symptoms of a stroke. I pointed out the likelihood of a pelvic fracture, but the doctor did not pay attention. After the patient was transferred several times, the doctor ordered the patient to be transferred to the orthopedic service; on the day of admission, the doctor did not listen to us and injured the patient. When we object to this, they get angry.”

On the other hand, deficiencies in leadership and coordination in the health care team lead to weakness in interdisciplinary care, making the MNC more likely. “The nurse is a member of the care team; the team should have a coordinator and all members should work together and the patient’s preferences should be taken into account. There is no such thing,” said a nurse with 20 years of experience (Nurse 2).

Several nurses felt that limited access to interdisciplinary specialists could lead to MNC. The lack of specialist nurses, physiotherapists, dietitians, occupational therapists and social workers makes it impossible for patients to benefit from specialist staff and their needs are neglected. “Many frail older adults need physiotherapy, occupational therapists, or social help to get back into the community, but many of them are homeless. We actually know our patients need it, but we really can not do anything about it,” said a nurse with four years of experience (Nurse 5).

##### Clinical incompetence

Caring for the frail elderly is a complicated task that requires expertise. This complexity arises from multiple and concurrent chronic illnesses, the use of multiple medications, and the possibility of complications in the hospital. The lack of knowledge, skills, and experience of nurses in dealing with frail elderly patients leads them to be unaware of the problems these patients face. A nurse with an MSN degree and eight years of experience commented (Nurse 9): “Novice nurses do not know that they should follow up on the frail older adult’s medications that he is taking at home. For example, an older adult is admitted to the hospital for a fractured pelvic and does not remember his medications. The nurse forgets to track what he’s taking, so the patient does not take the blood pressure pill, blood pressure rises to 20 or 24, and he suffers a brain hemorrhage.”

##### Weakness in continuing education

The content of nurses’ continuing education programs is determined according to up-to-date medical priorities, and they do not cover the clinical training demands such as caring for the frail elderly. “Most of the teaching materials are related to diseases, such as the management of hypertension, diabetes, and special diseases. As far as I know, we do not have special training for aging,” said a nurse with 28 years of experience (Nurse 7).

In addition, the training methods are not proportionate to the needs of the nurses. Due to the busy shift schedule, it is difficult for them to attend the training program in person. Distance education is not designed and delivered according to standard principles. As a result, participants are reluctant to take continuing education courses and attend these classes to earn annual continuing education credits. “I have a long evening night shift, and I should attend the course tomorrow morning; I can not go, and if I have to go, I will not learn anything”. The online training is usually consists reading e-books, and I just answer the questions to get a good grade,” said a nurse with 20 years of experience (Nurse 17).

#### Frailty of the older adults

Physical and psychosocial problems of frail elderly lead to complicated and time-consuming care that increases the risk of MNC. This category of MNC exacerbating factors includes “physical health problems,” “ psychological health problems,” and “ weakness of social support.”

##### Physical health issues

Frail older adults suffer from multi-morbidity, polypharmacy and dependence in daily activities, which makes them need more attention in clinical care and exacerbate MNC. “They cannot manage most daily activities of life on their own and are often dependent,” said a nurse with 20 years of experience (Nurse 2).

##### Psychological health issues

Frail older adults are affected by various psychological problems such as depression or cognitive impairment such as delirium. A frail older adult with these problems is unable to properly communicate and express their problems and this leads to MNC. A nurse with 5 years experience said (Nurse 15), “My patient had severe bleeding in her stool, but she did not tell us. She generally communicates with difficulty, she is mostly depressed.”

Psychological problems affect adherence to treatment. Participants explained that any weakness in treatment adherence increases the need for nursing care and leads to MNC. A nurse with nine years of experience explained (Nurse 11), “Frail older adults are usually not cooperative. For example, one of them had her Foley catheter removed. They usually refuse to take their medications or become aggressive, which makes it difficult to work with them. It’s hard to work with these patients, and we do not have the time to deal with every one of their problems.”

##### Weakness of social support

Many frail older adults who are hospitalized, are uninsured and have no financial or social support. Low social support or homelessness can lead to prolonged hospital stays. The organization or family does not support these patients during hospitalization, and their primary care needs, which are usually met by families, are neglected. “Some frail older adults do not have a family caregiver to help them, and sometimes we do not come to help them. Sometimes their needs are ignored until someone like another family caregiver can help them. Some of them do not have a home when they are discharged from the hospital and they stay here so the social worker can find a place,” said a nurse with 20 years’ experience (Nurse 2).

### MNC moderating factors

The categories of “Support capabilities” and “ethical and legal requirements” are considered as moderating factors of MNC.

#### Support capabilities

MNC can be prevented by monitoring nursing care delivered at the patient bedside. This category includes the subcategories of “Supervisor support and supervision” and “Active family involvement.”

##### Supervisor support and supervision

Supervision and clinical support from nursing managers, such as staff nurses, head nurses, supervisors and nurse leaders can prevent MNC. A nurse with five years of experience said (Nurse 3), “Some of our supervisors do everything they can to solve problems and keep working.” Managers can focus nurses’ attention on the quality of nursing care and prevent MNC through performance-based evaluation. A nurse with 18 years of experience commented (Nurse 13), “When I come to the ward as a charge, I check everything at the patient’s bedside. For example, I assess which nurses have taken care of the patient’s bedsore. I act in such a way that all the nurses know that everything is carefully checked on the shift I am supervising. That way they deliver the care perfectly”.

##### Active family participation

The active involvement of the family is accompanied by their presence at the bedside. The patient’s family can prevent MNC by following the care process, meeting nursing needs, and providing for the patient’s well-being. A female nurse with eight years of experience said (Nurse 9), “A good and active family caregiver is very good for the frail older adult. For example, he moves him, gives him food, gives him medication, helps him to the toilet or bathroom, helps him eat, gives him high-fiber food, and gives the patient a sense of familiarity and trust.”

#### Ethical and legal requirements

Other MNC moderating factors include ethical and legal requirements, including the subcategories of “humanistic view of patient care” and “Concern for accountability.”

##### Humanistic view of patient care

Many nurses have a humanistic view of care and understand the positive and negative feelings of frail older people. This can dramatically mitigate the MNC. A female nurse with five years of experience said (Nurse 3), “Caring for the elderly reminds me of my mother, who was once in the hospital and preferred to be cleaned every day. So I force the nurse aid to bathe the frail elderly or wash their hands and face every shift”.

##### Concerns of being accountable

Many participants indicated that they were concerned about organizational accountability and its consequences, such as the reduction in scores due to incompetence labels. They believed that this issue is effective in preventing MNC. A nurse with 35 years of experience stated (Nurse 6), “If you do not do your job properly, you will be known on the ward as someone who does not take good care of the patients. So I am careful about caring for patients, especially frail older adults and patients without family caregivers.”

Several participants expressed concern about the legal response. They are so sensitive and careful when providing nursing care to avoid legal problems. “I am always aware that I can not leave care undone. If I encounter a problem, I take care of it quickly so that I do not get involved in legal problems later,” said a nurse with five years of experience (Nurse 15).

## Discussion

The present study was conducted to investigate the aggravating and moderating factors of missed nursing care (MNC) among hospitalized frail older adults using a qualitative approach. The results of the study showed that factors such as “age-unfriendly structure,” “inefficient care,” and “frailty of the older adults” could increase the risk of MNC. On the other hand, factors such as “support capabilities” and “ethical and legal requirements” will mitigate MNC.

One of the factors contributing to MNC in older adult care is inappropriate care environment. An inappropriate structure makes it impossible for nurses to meet the comprehensive needs of frail older adult patients. Previous studies of nurses working in US hospitals have also confirmed this finding [32, 33]. The results of the qualitative study by Ebright et al. indicated that all novice registered nurses who made medication errors were providing care in an unfamiliar, crowded work environment. The unsuitable environment of medical wards increases the workload, causing the nurse to lose control of the care, leading to MNC [32]. Therefore, age-friendly hospitals are designed to protect older adult patients. In these hospitals, the care process is managed so that older adults are not neglected [34]. However, achieving these structures requires extensive changes that low-income countries are unlikely to support [35]. It appears that designing appropriate models can assist low-income hospitals in ensuring the safety of frail older adults.

Another factor influencing MNC is the discriminatory behavior of nurses toward frail older adult patients who find it futile to care for an older adult. A study was conducted by Higgins et al. of nurses with 2 to 35 years of experience in a teaching hospital in Australia showed that negative attitudes can lead to disregard for the needs of older adults [36]. Nevertheless, neglect of futile care is a controversial issue. From an ethical perspective, there is no need to provide care if there is no evidence that the intervention will have a positive effect [37]. Futile care cannot be associated with the basic needs of older people. Moreover, futile care should be carried out together with the elderly patient, and awareness of his family and related legal issues should be clarified [38]. It seems that a comprehensive plan is needed to determine the boundaries between necessary and futile care.

Nurses are subjected to a high workloads due to high patient volumes, staff shortages, and the multitude of tasks that lead to MNC. Several studies have also confirmed these findings [14, 16, 17]. Although the high number of caregivers effectively prevents MNC, few nurses are employed due to the financial problems of the organizations and shortage of nurses. It is possible to make the best use of human resources by modifying the care process and reducing the workload in non-clinical nursing.

The present study showed that inadequate content of continuing education programs and improper implementation methods may contribute to MNC. Consistent with the present findings, the results of a systematic review by Rouleau et al. showed that e-learning has an impact on the knowledge and skills of clinical nurses [39]. Therefore, further studies are needed to design e-learning courses based on nurses’ educational needs, learning conditions, and motivations.

Physical and psychosocial problems and poor communication skills of frail older people exacerbate MNC. The results of a scoping review study by Fitzgerald et al. suggest that the complexity of care and the high demands of older adults mean that nurses are unable to implement standard nursing care [40]. Therefore, it is important to develop standardized clinical guidelines for nurses to meet the demands of caring for frail older adults and prevent MNC.

The present study also showed that frail older adults with low social and financial support are more vulnerable to negligence. These patients who do not have insurance support cannot afford medical expenses. Therefore, they turn to medical centers with delay, which leads to worsening of their clinical condition [41]. On the other hand, the lack of access to the medical history of these older adults may lead to complicated care and the occurrence of MNC, as well as an increase in the length of hospital stay and discharge problems [42].

Supervisor support and supervision may be useful in promoting nursing care and preventing MNC. Consistent with the finding of this study, Markey et al. explain in the review study that the supervisor can support the care process by enhancing clinical training, learning, and questioning [43]. In Iranian hospitals, clinical supervisors mainly focus on the written standards of the accreditation process. This limits their attention in evaluating clinical care. Therefore, nurses give priority to indirect tasks related to the accreditation process, which diverts their attention from direct clinical nursing care.

This study has shown that active family involvement in the care of frail elderly prevents negligence and improves the quality of nursing care. In the Iranian health care system, the involvement of family members in hospital care is common due to the shortage of nurses. A significant amount of primary care, such as moving, feeding, cleaning, bathing, toileting, dressing, and oral hygiene, is provided by the family [44]. Studies in Taiwan and Turkey have shown similar results regarding family involvement in care and its positive impact on the prevention of MNC [45, 46]. It seems that family cooperation in the care process increases the psychological support of older adults in the hospital setting, in addition to compensating for staff shortages. Moreover, their psychological support may prevent the exacerbation of frailty, which requires further studies.

According to the findings of the present study, nurses’ concerns about organizational and legal accountability may prevent MNC. The qualitative study by Dehghan-Nayeri et al. conducted on nurse managers with a BSN and at least 12 months of managerial experience also suggests that nurses’ responsive personality can mitigate the risk of MNC [47]. Thus, this phenomenon is not only due to external factors but also influenced by various intrinsic factors such as nurses’ personality. Educating nurses’ professional personalities can guide them to provide appropriate care and reduce the occurrence of MNC [48].

It is suggested that hospital managers can prevent MNC by implementing reforms in the environment and structure of nursing care, updating nurses’ knowledge and skills, and modifying the care process by reducing indirect tasks. In addition identifying frail patients, modifying nurses’ assessment toward a clinical practice-based approach, modifying error reporting processes, supporting the patient’s family’s involvement in care, and promoting the culture of humanistic care in the organization are recommended for hospital managers. It is also suggested that courses on the care of the frail elderly in the hospital be included in the nursing curriculum and continuing education. In addition, specific and multidimensional instruments are needed to determine the MNC in frail older adults. Some limitations of the study should be mentioned. First, the nurses had limited time to conduct the interview. Due to the heavy workload of the nurses, some of them could not attend the pre-scheduled interview appointments. Therefore, the researcher visited the ward several times on different days to interview them whenthey had enough time. Secondly, due to the limited transferability of the qualitative findings, the results of the present study may not be transferable to other populations with different cultural norms. Finally, although we sought to maximize the dispersion of participants, it is still likely that certain factors were neglected. Further research would therefore provide a broader range of experiences.

## Conclusions

Older frail people receiving hospital care are more likely to be affected by MNC due to high care needs, communication problems and complexity of nursing care. The present study suggests that factors such as inappropriate care environment, ineffective care and frailty of older adults contribute to MNC. On the other hand, support capacities and ethical and legal requirements could reduce MNC in for the care of these patients. Nursing managers can take practical steps to improve the quality of care by considering the aggravating and moderating factors of MNC. In addition, nurses with a humanistic view that understands the multidimensional problems of frail older adults and pays attention to their weakness in expressing needs can create a better experience for them in the hospital and improve patient safety.

## Data Availability

All interviews were transcribed into text files. The text files are kept in locked files and are only accessible to the first author. The datasets are not publicly available for privacy and ethical obligations, but dataset may be available from the corresponding author upon reasonable request.

## Abbreviations

MNC: Missed nursing care
CPR: Cardiopulmonary resuscitation
BSN: Bachelor of science in nursing
MSN: Master of science in nursing
PhD: Doctor of philosophy
COREQ: Consolidated criteria for reporting qualitative research
